# The single-suture technique for anterior cruciate ligament graft preparation provides similar stability as a three-suture technique: a biomechanical in vitro study in a porcine model

**DOI:** 10.1007/s00402-020-03350-5

**Published:** 2020-01-24

**Authors:** Jan Theopold, Stefan Schleifenbaum, Alexander Georgi, Michael Schmidt, Ralf Henkelmann, Georg Osterhoff, Pierre Hepp

**Affiliations:** 1 Department of Orthopedic, Trauma and Plastic Surgery, University of Leipzig, Liebigstraße 20, 04103 Leipzig, Germany; 2 ZESBO, Zentrum Zur Erforschung Der Stütz- Und Bewegungsorgane, Semmelweisstrasse 14, 04103 Leipzig, Germany

**Keywords:** Arthroscopy, Biomechanics, ACL reconstruction, Patient safety, Anterior cruciate ligament, Hamstring graft

## Abstract

**Purpose:**

Numerous techniques have been described for the tibial-sided graft preparation in anterior cruciate ligament (ACL) reconstruction. The use of less suture material for graft preparation is thought to improve ingrowth and to reduce the risk for infection. At the same time, the suture construct should be strong enough to resist the surgeon’s pull during tensioning of the transplant.

**Methods:**

In total, 39 fresh-frozen procine deep flexor tendons were used and prepared as four-strand grafts. In the three-suture group (*n* = 19), graft preparation was performed using three tibial-sided sutures, with each tendon end sutured separately. In the one-suture group (*n* = 20), a modified graft preparation using only one tibial-sided suture was applied. Each sample underwent load-to-failure testing (*N*_max_) after cyclic pre-loading. To estimate intraoperative tension forces acting on the tibial-sided suture constructs, the maximal tension force of 26 volunteers on such a construct was measured using a load cell.

**Results:**

The biomechanical testing of the two different suture constructs showed a significantly higher load-to-failure for the three-suture group (711 N ± 91 N) compared to the one-suture group (347 N ± 24 N) (*p* = 0.0001). In both groups, the mode of failure was a tear of the suture in all samples. A failure of the suture–tendon interface was not observed in any case. The median maximal tension force on the construct applied by the 26 volunteers was 134 N (range 73–182 N).

**Conclusion:**

The presented single-suture tendon graft preparation resisted to smaller failure loads than the conventional three-suture technique. However, no failures in the suture–tendon interface were seen and the failure loads observed were far beyond the tension forces that can be expected intraoperatively. Hence, the single-suture graft preparation technique may be a valuable alternative to the conventional technique.

## Introduction

Sufficient and stable graft fixation is one of the key elements for successful reconstructions of the anterior cruciate ligament (ACL) [1]. Initial stable fixation is essential to avoid elongation and failure before ingrowth of the transplant [2].

Different techniques for ACL reconstruction have been described [3, 4]. A well-established method is the use of the semitendinosus tendon [5]. In most cases of the single-tendon technique, the ACL graft is formed by a closed tendon loop, which must be secured with sutures at the ends of the graft [6]. The type of loop can vary and different techniques of folding the graft [7–9]. Securing its ends with sutures have been described and tested [10–12]. Most surgeons prepare their grafts with sutures on each end of the tendon. This requires time for preparation, and leaves the implanted graft with a lot of suture material inside the patient [12].

Different studies have shown that bioabsorbable and metallic screws have a higher failure rate than other implants [13]. This could be attributed to a loosening of the graft [14]. It is assumed that the interface between graft and screw is too weak in the early stage of osteointegration of the graft [15].

With 0.5%, surgical site infections are rare with ACL reconstruction, but they can be devastating when they occur [16]. Postoperative septic arthritis can result in intraarticular adhesions and scarring [16]. Implanted foreign material is a key risk factor for bacterial colonization and surgical site infection [17, 18]. In addition, a foreign body reaction may negatively influence healing and ingrowth of the tibial-sided graft. Hence, reducing the amount of suture material remaining in the patient’s body makes sense not only from an economic point of view [19].

The primary goal of this study was to compare the biomechanical strength of a single-suture tibial-sided ACL graft preparation technique to the conventional methods using three sutures.

The secondary goal was to evaluate the maximum tension forces acting on the suture construct intraoperatively.

## Materials and methods

### Samples

Forty porcine deep flexor tendons were prepared for the study. The tendons were harvested from the hind legs of 2-year-old domestic pigs and then fresh frozen and stored at − 83 °C.

The samples were thawed to room temperature for 12 h before the testing and cut to a length of 24 cm. Fresh-frozen porcine flexor tendons were used based on availability and comparable biomechanical properties compared to human tendons [20] and they had been established for similar testing setups in previous studies [21–23].

### Graft preparation

All tendon samples were prepared as four-strand ACL grafts by an experienced knee surgeon using #2 Fiberwire^®^ sutures (Arthrex, Naples, FL, USA).

**Fig. 1:**
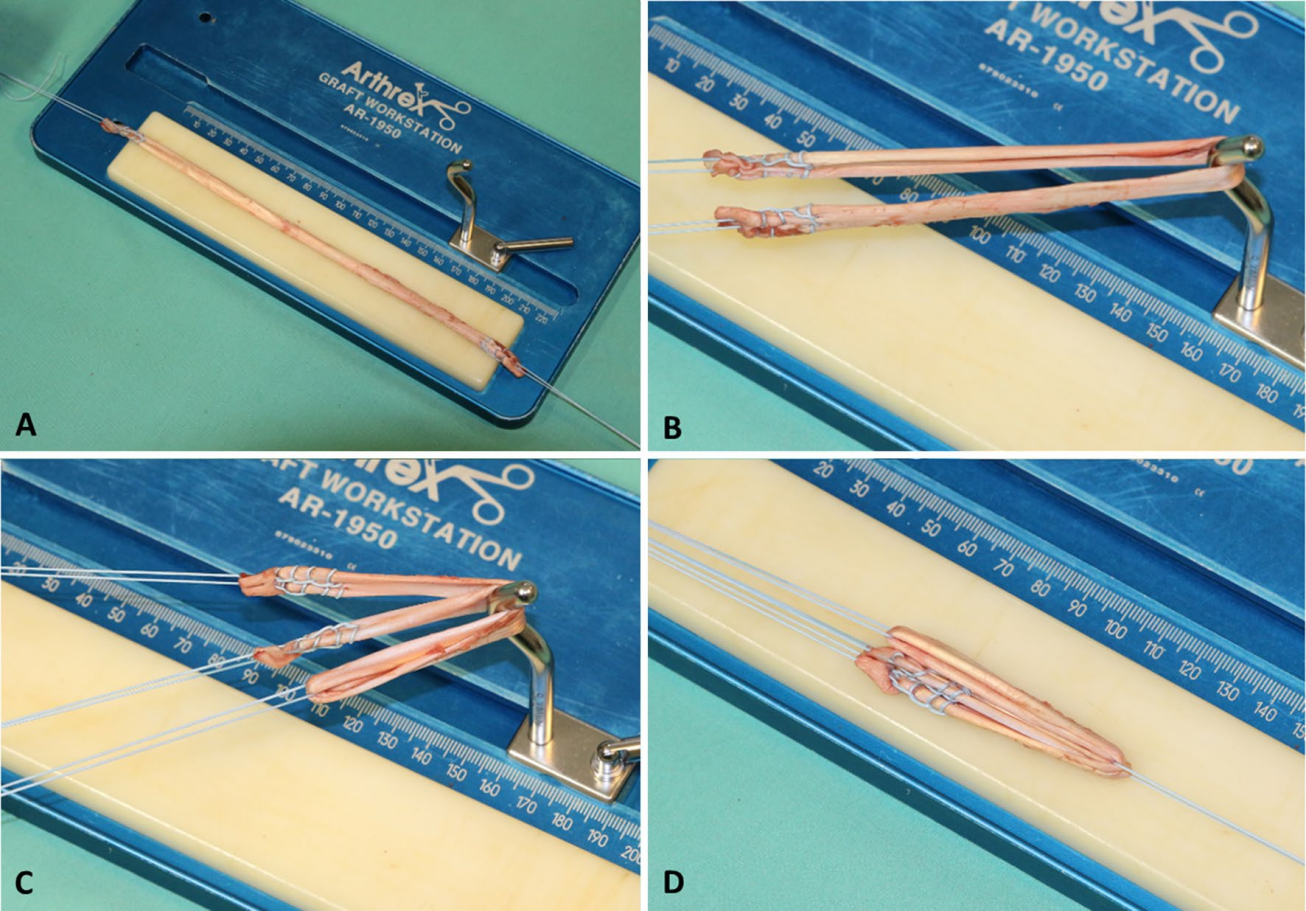
The conventional three-suture technique. **a** Separate suture fixation of each end of the tendon. **b** Forming a tendon loop. **c** Third suture pulled around the midpoint of the tendon. **d** Final four-strand graft with three tibial-sided sutures

In the three-suture group (*n* = 20), graft preparation was performed using three tibial-sided sutures: Each tendon end was sutured separately using Krakow stitches (four up and four down) and then a third suture was looped around the midpoint of the tendon to form a four-strand graft (Fig. 1).

**Fig. 2:**
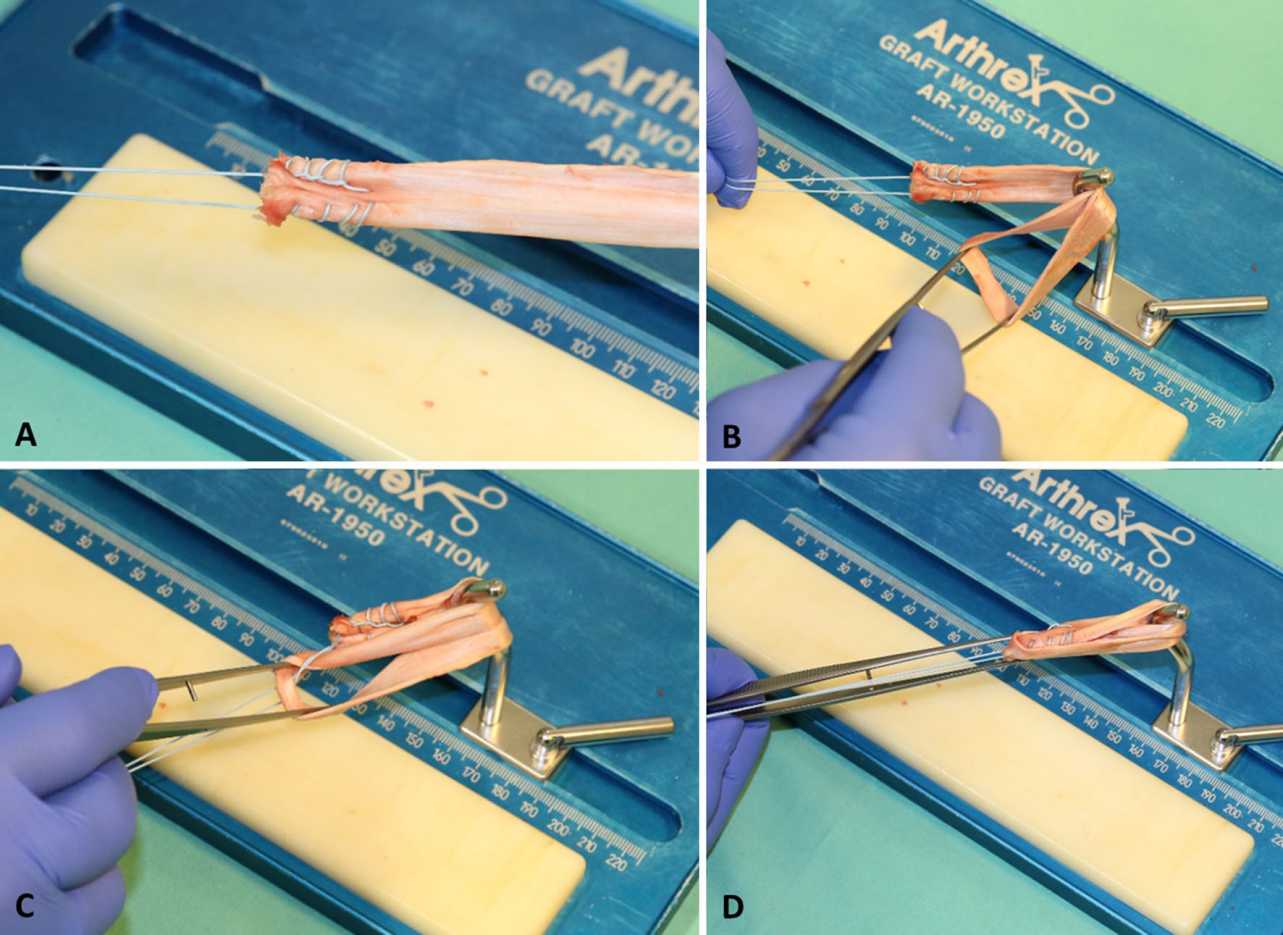
The one-suture technique. **a** Combined fixation of both ends of the tendon with one suture. **b** Forming a tendon loop. **c** Pulling the suture with the combined tendon ends through the midpoint of the tendon. **d** Final four-strand graft with one tibial-sided suture

In the one-suture group (*n* = 20), a modified graft preparation using only one tibial-sided suture was applied. Both tendons were sutured together, again using Krakow stitches (four up and four down). Then, the free suture ends were pulled through the midpoint of the tendon to form a stable four-strand graft (Fig. 2).

### Mechanical testing

**Fig. 3:**
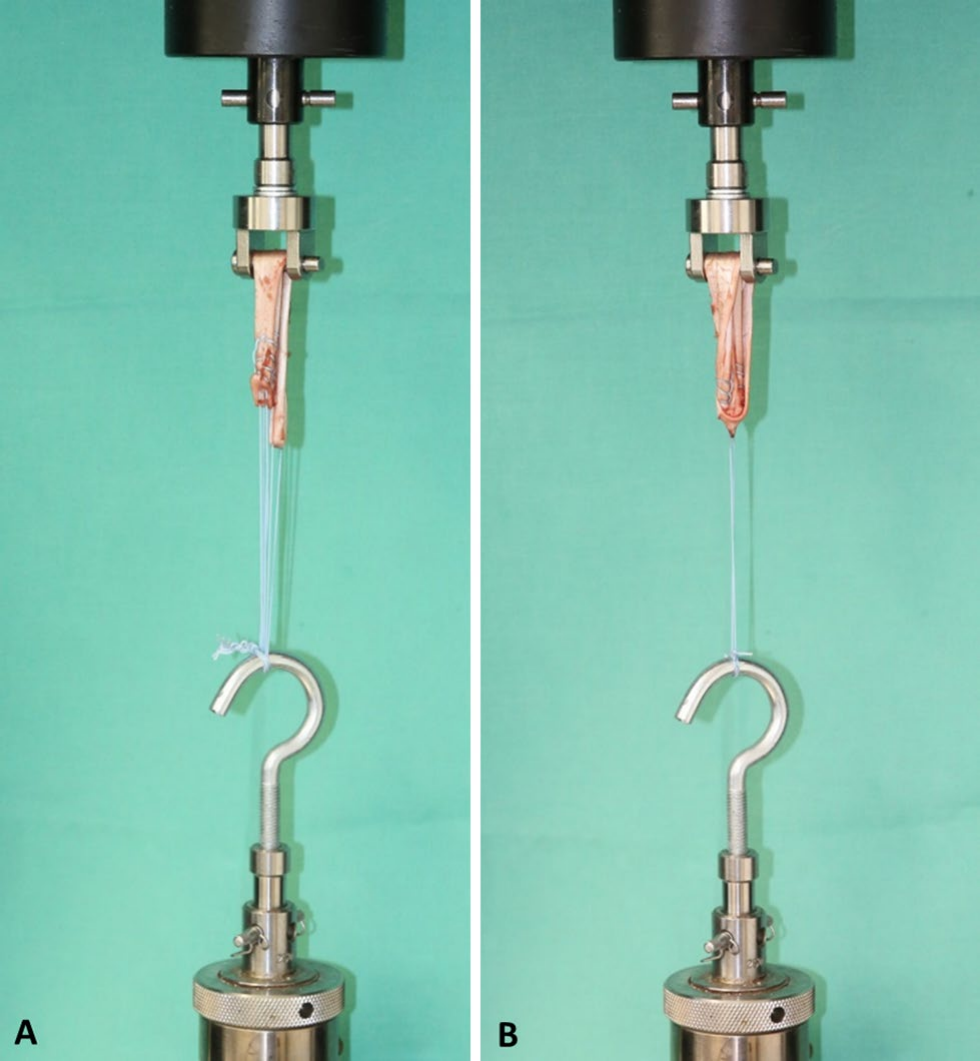
Testing setup. **a** Three-suture technique. **b** One-suture technique

After graft preparation, the samples were mounted into a mechanical setup on a standard testing machine (Typ 5566A, Instron, Norwood, MA, USA) with a hook and a customized hold fastener (Fig. 3).

After 50 sinusoidal cycles of pre-loading between 50 and 150 N at 1 Hz, a load-to-failure test was conducted at 20 mm/min.

### Surgeon’s tensile force

**Fig. 4:**
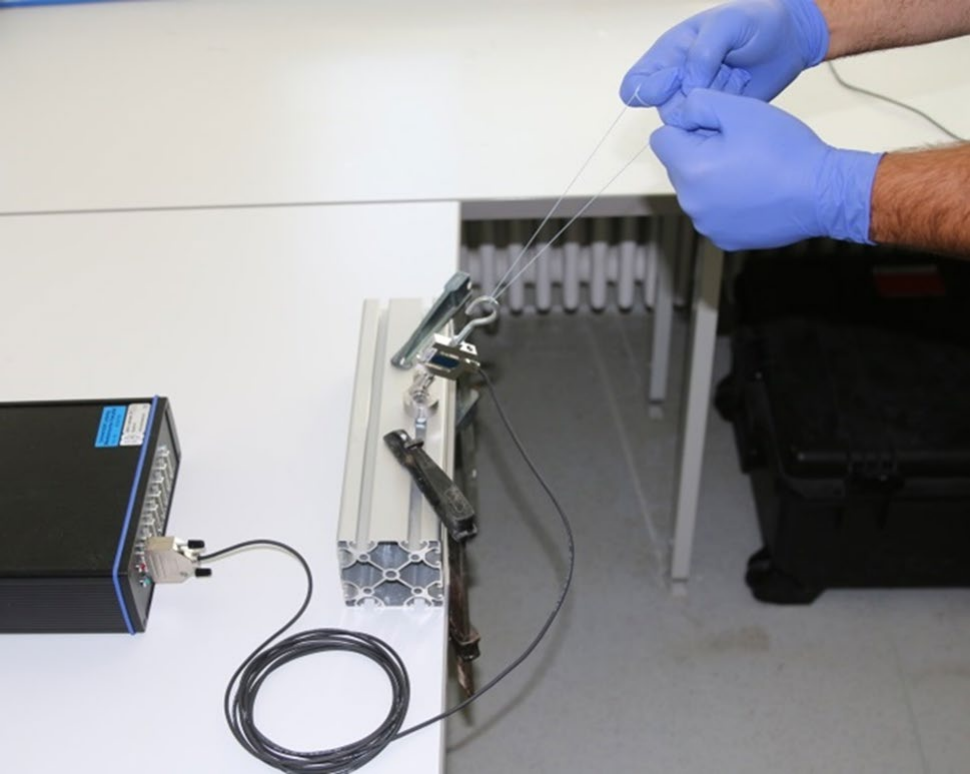
Setup for measuring the surgeon’s tension force. The suture is looped around a hook that is connected to a load cell

To evaluate the estimated force a surgeon can pull on an ACL graft during surgery and hence, to estimate evaluate the maximum tension forces acting on the suture construct intraoperatively, 26 volunteers were asked to pull on a suture/thread. The volunteers were orthopaedic residents and consultants (age mean 31 years, range 24–45 years; 6 female). The suture (#2 Fiberwire^®^, Arthrex, Naples, FL, USA) was looped around a hook to simulate the intraoperative position and angle during tibial tensioning of the ACL graft. Tension forces were measured using a tension force load cell (KD40s, ME-Messsysteme GmbH, Hennigsdorf, Germany) and recorded using a customized measurement software calculating the maximum tension force (Fig. 4).

### Statistical analysis

All data were collected in Excel 2013 (Microsoft Corporation, Redmond, WA, USA) for descriptive analysis. Further statistical analysis was done using SPSS V24.0 (IBM, Armonk NY, USA). A Mann–Whitney *U* test was used to compare differences in means between the two groups. The level of significance was defined as *p* < 0.05.

## Results

### Load-to-failure

**Fig. 5:**
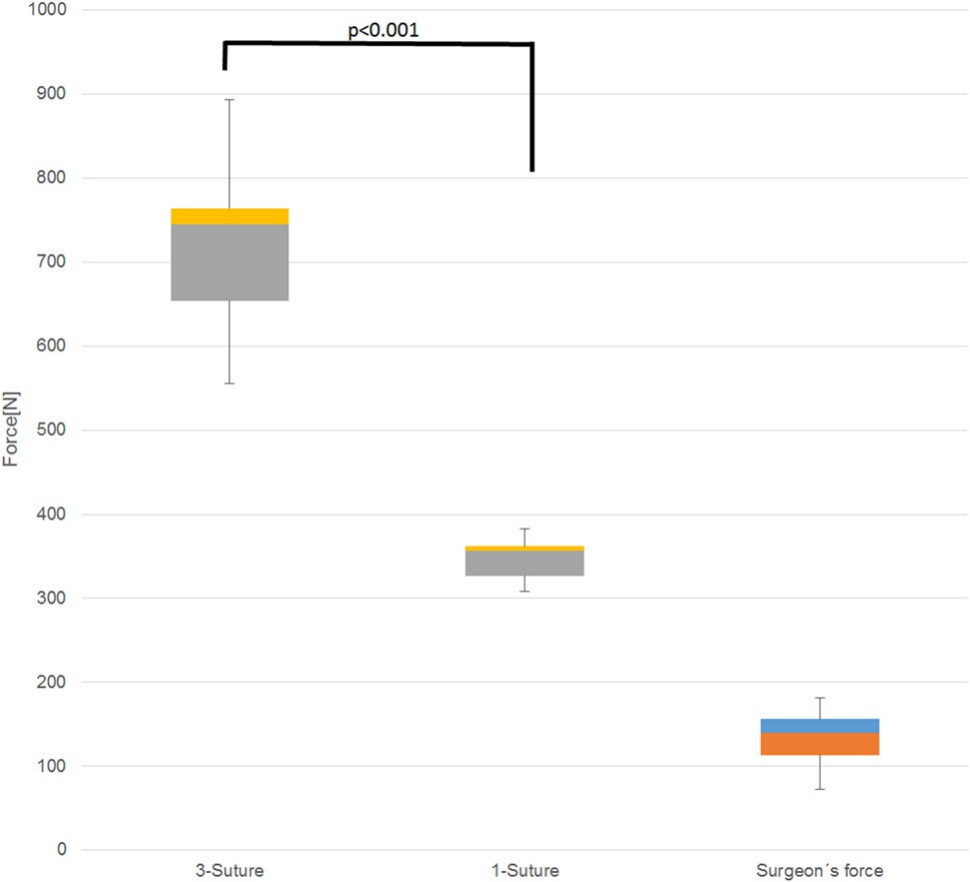
Boxplot graph showing failure loads and simulated surgeon’s maximum tension force

Mechanical testing was possible in 39 of 40 samples. One sample in the three-suture group had to be excluded from testing due to a pre-existing damage to the tendon. Thus, 19 grafts were tested in the three-suture group and 20 grafts in the one-suture group. In both groups, it was the sutures that always failed under load. No failure of the suture–tendon interface was observed in any case. The load-to-failure was significantly higher in the three-suture group (711 N ± 91 N) when compared to the one-suture group (347 N ± 24 N, *p* = 0.0001: Fig. 5). The values ranged from 555 to 892 N in the three-suture group and from 309 to 382 N in the one-suture group.

### Surgeon’s tensile force

During the simulation of tibial ACL graft tensioning, the 26 volunteers applied a mean maximum tensile force of 134 N ± 28 N (range 73–182 N; Fig. 5). For estimating the maximum tensile force, the volunteers used the same tensile force they would use in real tibial ACL surgery. As the key limiting factor, they reported pain in their fingers when pulling at the sutures.

## Discussion

The primary goal of this study was to compare the biomechanical strength of a single-suture tibial-sided ACL graft preparation technique to the conventional methods using three sutures.

The conventional technique using three sutures showed significantly higher failure loads. However, a failure of the suture–tendon interface was not seen in any sample, neither with the conventional nor the presented single-suture technique. In all cases, the failure of the construct was a failure of the suture(s). In a very similar biomechanical setup, Hong et al. reported a failure of the sutures in all cases, as well [12]. The manufacturer of the sutures used in this study declares a tensile strength of 300–345 N [24]. This is very close to the loads observed in the one-suture group of this study and is in line with the failure loads of biomechanical studies on different ACL graft suture preparations [12, 25]. In consequence, the difference between the two groups is based on differences in the sutures’ tensile strength and it seems very likely that it is solely the number of sutures that can tear that makes the difference.

In view of these numbers, it is of interest what the true forces are that may act on such a suture–tendon construct during surgery. In our simulation of tibial ACL graft tensioning, only one volunteer was able to achieve half the maximum tensile strength of the #2 FiberWire [24]. In fact, recent studies suggest that the true mean intraoperative loads during tibial-sided tensioning of the graft do usually not exceed 90 N [26–28]. In our simulation, some volunteers were injured by the #2 FiberWire, because it cut their fingers. Because of this, some surgeons try to prevent injuries by finger tapes [29]. This puts in question whether the found difference in failure loads between the two preparation groups is of any clinical relevance.

The limitations of this study include those inherent to a biomechanical in vitro study on porcine tendon. The forces applied were of only axial tension nature and do not take into account shear and torque forces that may occur during the ACL graft tensioning procedure. The samples were not human semitendinosus or gracilis tendons as used for ACL reconstructions but deep hind leg flexor tendons obtained from pigs. The influence of drying, autolysis and freezing of the tenons during the harvesting procedure and storage on their biomechanical behavior remains unclear and hence, limits the application of our findings to living tissue [30].

However, Domnick et al. could demonstrate that for biomechanical investigations, fresh-frozen porcine flexor tendons represent an appropriate substitute for human semitendinosus tendons [20]. Both porcine flexor tendons and the mechanical setup in this study have been used in other investigations on the strength of suture–tendon constructs [12, 20–23]. Freezing the samples for storage might have influenced the material properties, but the tensile force gets lower because of freezing [20]. This effect only supports our statement positively.

A limitation of the surgeon’s tensile force simulation is the fact that no pulling aids like clamps or forceps were used. Such the factor limiting the tensile force of the volunteers was often pain from the thread cutting into the fingers [29].

In summary, however, ACL graft preparation with the presented single-suture technique seems sufficiently stable in view of the tensile forces that can be expected intraoperatively. Even though based on in vitro observations, the results of this study may serve as basis for future clinical studies on this topic.

## Conclusion

The presented single-suture tendon graft preparation resisted to smaller failure loads than the conventional three-suture technique. However, no failures in the suture–tendon interface were seen and the failure loads observed were far beyond the tension forces that can be expected intraoperatively.

Hence, the single-suture graft preparation technique may be a valuable alternative to the conventional technique.
